# Approaching persistent pain and emotion dysregulation: Development of the hybrid emotion-focused exposure treatment

**DOI:** 10.1007/s00482-025-00885-7

**Published:** 2025-05-19

**Authors:** Sofia Bergbom, Hedvig Zetterberg, Ida Katrina Flink, Steven James Linton, Katja Boersma

**Affiliations:** 1 Center for Health and Medical Psychology (CHAMP), School of Behavioural, Social and Legal Sciences, Örebro University, 70182 Örebro, Sweden; 2 Department of Psychology and Social Work, Mid Sweden University, Östersund, Sweden; 3 Department of Social and Psychological Studies, Karlstad University, Karlstad, Sweden

**Keywords:** Chronic pain, Emotion regulation, Psychological distress, Depression, Treatment implementation, Chronische Schmerzen, Emotionsregulation, Psychischer Stress, Depression, Behandlungsimplementierung

## Abstract

**Background:**

Persistent pain, one of the most common reasons for suffering and health care seeking, often co-occurs with emotional problems such as depression and anxiety. Within the Center for Health and Medical Psychology at Örebro University, Sweden, we have developed a new treatment aimed at addressing co-occurring persistent pain and emotional problems: hybrid emotion-focused exposure treatment. The overarching idea behind the treatment is that patients who struggle with comorbid pain and emotional problems need to develop skills in dealing with emotions as well as pain. With better skills in tolerating and soothing difficult emotions, patients will be more able to approach previously avoided stimuli and situations, such as movements, activities and social interaction.

**Objectives:**

This review aims to delineate the development of the hybrid emotion-focused exposure treatment. It begins by outlining the theoretical background, then proceeds to describe the techniques, discuss the evidence and conclude with an illustrative case example.

**Results:**

Thus far, the treatment has been tested in a single-case study and a randomized controlled trial with promising outcomes. Overall, the hybrid treatment seems to have a good effect on patients’ depressive symptoms and pain interference. The treatment is currently being implemented, and the implementation process evaluated, in primary and specialist care across Sweden.

**Conclusions:**

The hybrid emotion-focused exposure treatment seems to be a well-suited treatment for people with a high burden of persistent pain and emotional difficulties. There is good reason to implement the treatment in clinical practice and continue evaluating treatment effects across different contexts.

## Abstract (de)

**Hintergrund:**

Persistierende Schmerzen, einer der häufigsten Gründe für Beschwerden und Inanspruchnahme medizinischer Behandlung, treten oft gemeinsam mit emotionalen Problemen wie Depression und Angst auf. Am Center for Health and Medical Psychology der Örebro-Universität, Schweden, wurde eine neue Behandlung mit dem Ziel entwickelt, gemeinsam auftretende persistierende Schmerzen und emotionale Probleme anzugehen: die hybride emotionsfokussierte Expositionstherapie. Die übergreifende Idee hinter dieser Behandlung besteht darin, dass Patienten, die mit den Komorbiditäten Schmerzen und emotionale Probleme zu kämpfen haben, Fähigkeiten entwickeln müssen, um mit Emotionen und Schmerzen umzugehen. Mit besseren Fähigkeiten in Bezug auf die Toleranz und das Beruhigen schwieriger Emotionen werden die Patienten besser in der Lage sein, zuvor vermiedenen Reizen und Situationen, wie Bewegungen, Aktivitäten und soziale Interaktion, zu begegnen.

**Ziel:**

Ziel der vorliegenden Arbeit war es, die Entwicklung der hybriden emotionsfokussierten Expositionstherapie zu skizzieren. Zu Anfang wird der theoretische Hintergrund dargestellt, dann geht es weiter mit den Techniken sowie der Erörterung der Evidenz, und schließlich folgt ein anschauliches Fallbeispiel.

**Ergebnisse:**

Bisher wurde die Therapie in einer Einzelzentrumstudie und einer randomisierten kontrollierten Studie mit vielversprechenden Ergebnissen untersucht. Insgesamt scheint die hybride Therapie eine gute Wirkung auf die depressiven Symptome der Patienten und den Einfluss von Schmerzen zu haben. Derzeit wird die Therapie in der Primär- und der spezialisierten Versorgung in ganz Schweden implementiert und der Implementierungsprozess evaluiert.

**Schlussfolgerung:**

Die hybride emotionsfokussierte Expositionstherapie scheint eine gut geeignete Behandlung für Menschen mit einer hohen Belastung durch persistierende Schmerzen und emotionale Schwierigkeiten zu sein. Es gibt gute Gründe, die Therapie in der klinischen Praxis zu anzuwenden und Therapieeffekte in verschiedenen Kontexten weiter zu untersuchen.

Persistent pain is a global phenomenon, with millions of sufferers worldwide. About one fifth of the population report chronic, disabling pain [4] and pain is the most common reason for seeking health care [24]. Many approaches to treat chronic pain have been developed, including surgical, medical, and psychosocial approaches [22]. However, long-term treatment effects are moderate at best and despite advances in the past decades many have a lifelong struggle with pain [6]. There is consensus in the field that pain and emotions often co-occur [23] and that negative emotions may play an important role in the development and maintenance of a persistent pain problem. Therefore, the hybrid emotion-focused exposure treatment (Hybrid) project at Örebro University, Sweden aimed at addressing comorbid pain and emotional problems through an emotion-focused psychological treatment. The target population for this treatment is individuals suffering from chronic primary pain, specifically persistent musculoskeletal pain of a duration of more than 3 months that is accompanied by substantial daily interference and distress. For this condition, introduced in ICD-11, pain is conceived as a disease in its own right, and not a symptom secondary to underlying disease processes [27]. Mechanistically, the persistence of pain is thought to be predominantly nociplastic and due to central sensitization processes [12].

While many of the available treatments include interventions specifically targeting psychological distress, they are not always successful. For example, several multimodal and psychological interventions have integrated stress management and relaxation techniques to target high levels of distress. Yet, there is evidence that those with the highest levels of psychological distress, i.e., with stress-related symptoms, depression, and anxiety, still struggle after treatment and have worse outcomes than those with lower levels of psychological distress [28]. Moreover, many interventions have been developed aiming to target “everything for everyone”, missing the important point of treatment tailoring and person-centered care [10, 17, 33]. In this article, we will discuss a treatment approach to better link our interventions to theoretical advances and address comorbidities in people suffering from comorbid pain and emotional problems.

## Theoretical background

In the early 2000s, Vlaeyen and Linton proposed a framework for understanding the development of chronic pain problems: the fear and avoidance model [31]. Since then, the model has gained much interest and resulted in massive experimental as well as clinical development [21]. The fear avoidance model sheds light on how a fearful response to pain may lead to avoidance of the movement or activity predicting the pain—especially if the pain has a high threat value and is paired with catastrophic thinking. The avoidance of movement and activities in turn risks fostering both depressive symptoms and disability—leading to more pain. The model points out several important psychological mechanisms, especially protective behaviors such as avoidance, driving the development from acute to persistent pain.

A prominent clinical example of theory-based treatment development resulting from the fear avoidance model is the adaptation of exposure in vivo to pain-related fear and avoidance behaviors [29, 30]. Exposure in vivo specifically targets both beliefs of the threat value of pain, fear of pain, and avoidance behavior [5, 21]. While exposure in vivo has demonstrated to be effective for some, others have less successful outcomes. Interestingly, it seems like those who catastrophize the most about their pain may benefit little from the treatment [7], potentially due to difficulties in downregulating the negative emotions triggered by exposure. Hence, the fear and avoidance model might need to be complemented by a theoretical understanding of emotions and emotion regulation in order to improve treatment outcomes of exposure in vivo among people with comorbid pain and emotional problems.

**Fig. 1:**
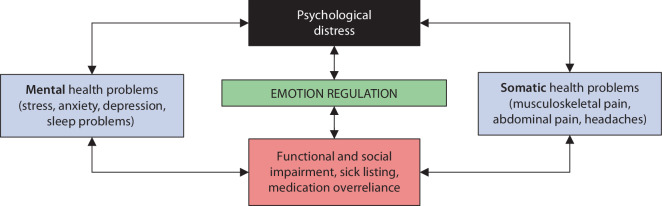
A transdiagnostic view of the relation between mental and somatic health problems. (Adapted from [25])

An important key to solving the problem of frequent comorbidities is to develop a more integrated conceptualization of, and treatment model for, patients with concurrent pain and emotional distress. Current advancements in the fields of emotion science and clinical psychology may provide direction. One way to understand the co-occurrence between mental and somatic health problems is offered by the ‘transdiagnostic’ perspective [2, 25]. Rather than viewing pain and emotional problems as separate disorders that are also treated separately, the transdiagnostic approach focuses on shared underlying mechanisms. In fact, pain and emotions share many similarities [14]. This perspective focuses on key emotion regulation processes that maintain and contribute to the exacerbation of both mental and somatic symptoms such as pain (Fig. 1; [26]). With respect to stress, anxiety, depression and pain this means that, instead of the one being a consequence of the other, the co-occurrence is explained by the (over)activation of basic emotion regulatory processes such as worry, rumination, emotional suppression and avoidance behaviors.

Identifying these shared mechanisms in turn offers the benefit of a treatment that addresses both the pain and the emotional problems, thus, being more effective and parsimonious. Based on these theoretical developments, we have systematically developed a new treatment model that specifically targets underlying emotion regulation problems. This hybrid emotion-focused exposure treatment (termed ‘hybrid’ treatment hereafter) combines exposure methods based on the fear avoidance model with an emotion regulation approach informed by procedures in dialectical behavior therapy (DBT). DBT is a treatment approach, originally developed for chronically suicidal patients but thereafter successfully adapted for patient groups with other complex problems characterized by high degrees of emotional dysregulation. It centers on teaching patients emotion regulation skills in a context of nonjudgmental acceptance and desired goal pursuit. Patients are trained to accurately identify, understand, soothe, and effectively act upon their emotional responses to aversive stimuli by means of a range of techniques [11].

The hybrid emotion-focused treatment integrates procedures from exposure and DBT with a clear uniting conceptualization focused on targeting underlying processes that maintain co-occurring pain and emotional problems. The term hybrid thus refers to the combination of these two theoretical frameworks and treatment approaches.

## The hybrid treatment

### Techniques

The hybrid treatment is a manual-based, principle-oriented intervention [16]. It is divided into four stages, and advancement from one stage to the next is based on achievement in the previous stage. There is no set number of sessions for each stage—the length of each stage is adapted to the patient’s needs. Throughout all four stages, the patient is encouraged to develop important skills to deal with difficult emotions as well as pain. In this way, the specific objectives for each of the stages build upon each other.

The objective of the first two stages is to prepare the patient for successful exposure. In the *first* stage, the therapist and the patient build a working relationship, strive to soothe distress, and develop relevant goals. Moreover, a focus is on becoming an “avoidance detective” for identifying triggers for emotion and pain that result in emotional or physical avoidance. The identification of the specific situations and stimuli that provoke negative emotions and pain that lead to avoidance for an individual patient is crucial for developing a hierarchy for the exposure treatment.

The objective of the *second* stage is to build skills to afford exposure. During this stage, skills that will help the patient move toward their goals are identified and practiced. These typically involve tolerance skills (e.g., to tolerate an increase in pain or emotion), soothing skills (e.g., self-validation, relaxation), and change skills (e.g., problem solving).

The overarching objective of the remaining two stages is exposure to enable approaching triggers and achieving personal goals. In the *third* stage, exposure for avoided movements and emotions is initiated. By applying the skills learned in the first stages of the treatment, patients learn to approach previously avoided situations which in turn reduces fear, worry, and catastrophizing. While some situations may focus on movements only, many of the situations that the patient avoids involve both movements and difficult emotions. Exposure to difficult emotions can be realized through bringing the patient in contact with previously avoided emotion-provoking situations, such as social situations that provoke fear. It can also be realized through talking about situations that have provoked difficult emotions, for example, feeling anger and guilt when having to ask a family member to do a chore that the patient cannot do due to pain. Between sessions, the patient and therapist develop homework assignments to practice this in their everyday life. The *fourth* and last stage has a specific focus on social situations, in line with the transactional model of emotion regulation [8]. The objective is to build skills for emotion-provoking social situations. During this stage, the patient is encouraged to use validation and context sensitivity to identify when, with whom, and how to disclose their experiences, emotions, and pain. These skills are particularly important when the patient needs to interact with others, such as family and loved ones but also health care professionals and colleagues at their workplace.

The objectives, along with the specific therapeutic techniques that therapists are encouraged to use are summarized in Table 1.

**Table 1:** Objectives and therapeutic techniques in the four stages of the hybrid emotion-focused exposure treatment

Stage	Objectives	Therapeutic techniques
Stage 1	Working relationship, soothe emotions, develop relevant goals	Self-monitoring and behavioral chain analysis
		Validation
		Dialectic stance
		Metaphors and short psychoeducation
		Goal-setting and valued commitment
Stage 2	Build skills to afford exposure	Psychoeducation
		Goals: solution analysis
		Skills training
		Metaphors and dialects
Stage 3	Exposure for movements and emotions	Exposure in vivo: avoided movements
		Exposure in vivo: avoided emotions
		Generalize exposure skills
		Exposure experiments
Stage 4	Build skills for emotion-provoking social situations	Identify people and topics that elicit negative
		affect/avoidance
		Listening, validation, honest messages
		Identify cues for communication
		Disclosure
		Apply regulation skills to implement in vivo

The manual describes therapeutic techniques that are designed to increase the probability that the patient will reach the objectives of each stage [16]. In the first and second stages, the therapist uses behavioral analysis, validation, and psychoeducation. Detailed behavior analysis will allow for the therapist and patient to identify avoided stimuli that act as triggers for negative emotions and pain. The therapist’s use of validation will facilitate building a working alliance, and at the same time act as model behavior for the patient’s own self-validation later in the treatment. The point of psychoeducation is to develop an understanding of how emotions and pain may interact and mutually maintain each other. The therapist also uses metaphors and communicates a dialectic stance, both with the purpose of increasing perspective taking and flexibility. Towards the end of the second stage, the patient is encouraged to practice soothing and tolerance skills in order to prepare for exposure to emotion- and pain-provoking stimuli.

At this point, a form of graded exposure in vivo to previously avoided movements, emotions, and situations becomes the focus of treatment. This involves the use of “exposure experiments” that test the patient’s hypotheses about the outcome of doing certain movements or activities. This is learning by doing in order to assist patients in approaching previously avoided activities and situations while using the coping skills they have learned to achieve this. Since social situations are of particular importance, the final stage focuses on dealing effectively with difficult ones. For example, a patient may practice communicating about difficult topics, without resorting to avoidance and emotional dysregulation.

### Evidence

The hybrid treatment has been developed and refined step-by-step. First, it was evaluated using a single-case experimental design with six participants who were closely followed, acting as their own control [15]. The outcomes were very promising, showing that all participants improved on key variables such as function, pain catastrophizing, acceptance and negative affect. As many as five of the six participants showed clinically significant improvements across all outcome measures while the sixth participant showed some improvement. Hence, there was reason to follow up the single-case study with a randomized controlled study.

In 2019, Boersma et al. performed a larger scale randomized controlled trial comparing the hybrid treatment to standard cognitive behavior therapy (iCBT) as an active control condition [3]. The study population was individuals with persistent and disabling musculoskeletal pain not emanating from malignancies (e.g., cancer pain), systemic diseases (e.g., rheumatoid arthritis) or localized single-joint osteoarthritic conditions in the lower extremities. Self-reported diagnoses included, for example, chronic low back pain, fibromyalgia, and whiplash. In addition, individuals had high levels of emotional distress operationalized as ≥ 8 points on one of the subscales of the Hospital Anxiety and Depression Scale. After inclusion, participants were also screened using MINI (Mini International Neuropsychiatric Interview) which showed that two thirds of the sample fulfilled criteria for a clinical diagnosis for major depressive disorder and/or an anxiety disorder.

Among the 115 included participants, those who received the hybrid treatment (10–15 sessions over 16–21 weeks) reported significantly less catastrophizing and pain interference at posttreatment and significantly less depression and pain interference at follow-up, compared to those who received standard CBT. The participants, however, did not differ in terms of anxiety and pain intensity. Moreover, analyses of clinically significant improvement favored the hybrid treatment, but these effects were not statistically significant. The authors concluded that the hybrid treatment should be considered “an acceptable, credible, and efficacious treatment option” for people with comorbid pain and emotional problems [3]. Thus, this trial strengthened the efficacy of the hybrid treatment and justified an effectiveness evaluation of the treatment in clinical practice to study whether the treatment can be successfully implemented [1]. While still awaiting the results of the effectiveness evaluation, a process evaluation studying implementation obstacles has been finalized [19]. In this study, we investigated how psychologists experienced using the hybrid treatment in their own clinical setting. The psychologists had been trained to perform the treatment and thereafter took part in the effectiveness trial by recruiting, assessing and treating 2–5 patients each in their own clinic, throughout Sweden. They were invited after the trial was completed to share their thoughts and experiences about the implementation of the treatment, in a semi-structured interview. The results showed that the psychologists could identify both factors that facilitated and that hindered the implementation. Facilitating factors were the presence of engaged clinic leadership, having a clear mandate to treat the target patient group, and a well-established collaboration with other health care professionals. Hence, results suggest that when planning to implement a new treatment such as the hybrid treatment in clinical practice, a large portion of the effort needs to be directed to the organization within which the implementation will take place.

## Case example

The case example below illustrates the hybrid treatment, with 29-year-old M, a female patient. The example is a fictional case, integrating clinical experience.

### Description

M has suffered persistent back pain for 12 years beginning at age 17. Her general practitioner (GP) said that it was due to menstruation. The problem, however, became worse and she was sent to orthopedics where she received several examinations and three MRIs over the course of a few months and was eventually diagnosed with a herniated disc and instructed to rest, avoid lifting, twisting and heavy work. M has been off work frequently and is currently working half time. Despite treatments, the pain progressed and became persistent which is why a psychologist was asked to assess and treat her.

M works for an information technology company; she works hard, enjoys the work, and is well liked and respected by her employer. Yet, she has great difficulty working because of the pain. The company has provided ergonomic help and the possibility to work at her own pace, but this has had no effect. Current tests show no organic abnormalities or “red flags”. She has done many courses of physical therapy with no improvement. Her doctors say there is nothing more they can do but provide pain medication and sick listing which M says are of little help.

The pain has greatly impacted on M’s physical function. She frequently rests and always avoids walking or biking for more than 10 min, does almost no housework, shopping or the like for fear of exacerbating the injury. Earlier M was active in sports and an avid hiker doing weeklong hikes and camping with her partner. She was also very active socially. During the assessment M says that she feels depressed and anxious and is extremely worried about her future. She is afraid she may lose her job, her career development, and her partner. M shows clear clinical signs and has high scores on fear and avoidance, anxiety, catastrophizing, and depression inventories.

### Treatment focus

Since M is avoiding movements and has considerable emotional difficulties the treatment plan focused on emotion regulation skills training and exposure in vivo a la the hybrid. Abundant validation was provided to sooth M’s negative affect, and she was then taught self-validation and other strategies to regulate her emotions. At the same time, a crucial aspect was to develop personally relevant goals. This was facilitated by M’s clear interest in developing her relationships, activity level and career. To increase physical functioning, exposure in vivo was started in session. This centered on confronting M’s thoughts and beliefs that she would further injure herself. Homework was oriented on re-establishing physical function (behavioral experiment: bike 12 min), her relationship with her partner (planning together and do an activity: go for a 1 km walk), and her work (contact workplace: gradually increase work time), her social life (invite friends for a coffee at a favorite café).

At the psychologist’s initial assessment, M moved reluctantly, and was quite distressed, crying frequently. However, she responded to validation and was engaged in developing her goals which she said induced hope. She understood avoidance and could identify triggers. Consequently, exposure led to large improvements in physical function. Homework focused on social situations at home and at work. M was diligent in doing the homework and discovered that she was able to participate in many desired physical and social activities at home and work. Although much more active, M reported a significant decrease in pain as well. She achieved her planned goals. At follow-up, M said that a barrier (the fear etc.) had been removed resulting in a meaningful improvement in her life.

## Discussion

Developing new, effective psychological treatments is a complex interplay among theory, empirical data, and practice. Our point of departure was people struggling with chronic (> 3 months) and interfering pain not primarily stemming from underlying disease processes or (nerve) damage, and comorbid high levels of depressive and anxiety problems, who often do not respond satisfactorily to available treatments. We used a transdiagnostic approach based on the fear and avoidance model and the transactional model for emotion dysregulation [8, 32] to identify probable shared underlying mechanisms to both problems. This resulted in the hybrid treatment which tackles emotional and persistent pain problems at the same time, using methodology from CBT (exposure) and DBT (validation, dialectics, emotion regulation skills training). The idea of combining a focus on emotions and pain in a single treatment package is shared by others. For example, Lumley & Schubiner developed the emotional awareness and expression therapy (EAET) combining exposure-based interventions with intensive short-term psychodynamic therapy and experiential therapies [18]. While the hybrid treatment rests on the transdiagnostic notion of emotion dysregulation driving the pain problem and emotion regulation skills training as key mechanism for change, EAET focuses on recognizing, disclosing, and processing trauma and conflict as key to accessing more adaptive emotions. The development of the hybrid treatment and development of EAET are hence two different examples of the interplay, guided by theory and in the process of gathering evidence and changing clinical practice.

The hybrid treatment takes a transdiagnostic approach with an orientation towards shared mechanisms that maintain both the pain and the emotional difficulties [25]. Several authors have called for new approaches that focus on a process-based rather than syndrome-based approach [9], not least for pain [20]. The hybrid treatment heeds this call by addressing transdiagnostic mechanisms, for example, emotion (dys)regulation.

Overall, the hybrid treatment bears promise, though future research is needed to answer important questions about effectiveness and implementation. First, we need more studies from different settings to assess effectiveness. While the evidence so far indicates that the effects are better than standard CBT for people suffering from comorbid pain and emotional problems, this is based on two studies from our group. Second, is the need to study how the treatment might be implemented into standard care. The treatment is usually conducted by a trained psychologist (sometimes in collaboration with a physical therapist) and these are not always readily available. Moreover, the manual requires many therapeutic skills, and training to competency is needed for successful implementation [17]. Clinically, we have observed a tendency to stay in the preparatory stages (stage 1–2) and hesitating to move forward to the exposure stages (stage 3–4), possibly associated with a weaker treatment effect. Whether this tendency is due to therapist and/or patient factors is a subject in need of further study. That exposure is difficult for therapists to perform and subject to ‘drift’ is a finding that is well corroborated in the literature [13] and might be a specific risk, or challenge, for therapists delivering the treatment.

Consequently, an important question is how well the intervention can be implemented into usual clinical practice. While our preliminary findings suggest that there are factors related to successful implementation, such as mandate, collaboration and leadership support, the question is whether the effects translate to new contexts. Will the format suit different therapists? Will the design of the intervention fit within patient flows in different clinical settings? Will the treatment adherence be affected when treatment is offered in regular clinical care? Another question, related to the implementation into standard care, is how well the treatment can be adjusted to fit different organizational needs. The treatment has not yet been tested with groups of patients, and while there may be some advantages to the group format, the individual analysis is key to identify maintaining mechanisms that need to be addressed in treatment. Hence, if tested in a group format the individual analysis of the co-occurring pain and emotional distress might need to be left intact. The ongoing implementation study will provide some answers and direct future development and research, but more implementation research is desired.

It is our hope that the hybrid emotion-focused exposure treatment will contribute to improvements in the care for people with pain and concurrent emotional problems. We look forward to future studies that will improve its content and implementation.

## Practical conclusions

- People suffering from co-occurring persistent pain and emotional distress are present in primary as well as specialist care and oftentimes struggle to find effective treatments.
- The hybrid emotion-focused exposure treatment is specifically developed to address mechanisms that are associated to this co-occurrence of pain and emotional distress.
- The treatment is a ‘hybrid’ of dialectical behavior therapy and exposure.
- The main components of the treatment are validation, skills training to soothe emotions, goal setting and valued commitment, and exposure for movements and emotions.
- The treatment is promising, decreases depressive symptoms and pain interference, and is being implemented in both primary and specialist care across Sweden.
- One challenge is that the treatment demands specific therapist skills related to exposure, and a need for training and supervision.
- The hybrid treatment is a suitable option for a burdened patient group who suffer physically and emotionally.

## Declarations

For this article no studies with human participants or animals were performed by any of the authors. All studies mentioned were in accordance with the ethical standards indicated in each case.
